# The nature and fate of natural resins in the geosphere XIII: a probable pinaceous resin from the early Cretaceous (Barremian), Isle of Wight

**DOI:** 10.1186/1467-4866-9-3

**Published:** 2008-01-29

**Authors:** P Sargent Bray, Ken B Anderson

**Affiliations:** 1 Department of Geology, SIU Carbondale, Carbondale IL, 62901, USA

## Abstract

Terpenoid resin is produced by all families and most genera of the order Coniferales (the conifers), and the distribution of terpenes present in most conifer resins is characteristic of the originating family. Analyses of early Cretaceous (Barremian) amber (fossil resin) from the English Wealden, Isle of Wight, southern England, by pyrolysis-gas chromatography-mass spectrometry (Py-GC-MS), indicate a terpene distribution dominated by abietane- and labdane-type terpenes. Similar distributions are observed in some species of the extant family *Pinaceae*. The Pinaceae are well represented within the Wealden deposits of southern England, by only one (known) species, *Pityites solmsii *(Seward) Seward, whereas the macro-fossil record of these deposits is dominated by the extinct conifer family *Cheirolepidiaceae*, for which no resin chemistry has been reported. By analogy with modern materials, it is probable that the ambers found in these deposits are derived from an extinct member of the *Pinaceae*, but given the absence of evidence concerning the chemotaxonomy of the *Cheirolepidiaceae*, this family cannot be excluded *a priori *as a possible paleobotanical source. These ambers may therefore be assigned to either the *Pinaceae *or to the *Cheirolepidiaceae*. These samples are the oldest ambers to date to yield useful chemotaxonomic data.

## Background

The Isle of Wight, off the south coast of England, is well known for rich lower Cretaceous (Barremian) fossil beds. In addition to numerous fossil fauna, (the Isle of Wight is widely recognized as one of the most important dinosaur fossil sites in the world), extensive floral remains have also been reported. The paleoenvironment of these deposits has been characterized by Insole and Hutt [1] as semi-arid, with distinct wet and dry seasons. Based upon macrofossil evidence, Oldham [2]concluded that the paleoflora contributing to these strata was dominated by genera of the extinct conifer family *Cheirolepidiaceae*. Alvin et al. [3] further refined this assignment and have reported that the dominant conifer in this environment was *Pseudofrenolopsis parceramosa*, creating stands wherein *Brachyphyllum obesum *grew beneath the canopy. Infrequent examples of other taxa, including species assigned to the *Pinaceae, Araucariceae*, and *Taxodiaceae *(now considered part of the *Cupressaceae*), have also been reported in the Wealden [2,4,5], although the assignment of these early Cretaceous specimens to modern families has been challenged [5].

Almost all modern conifers produce resinous exudates. In extant species, these resins are dominated by di- tri- and/or tetracyclic diterpenes. Mono and sesquiterpenoids are also common constituents, and triterpenoids are present in some cases [6], but these are less important overall than diterpenoids in most conifer resins. Resins are also highly durable materials that are resistant to many of the biogeochemical processes that degrade other plant tissues and are, therefore, of considerable chemotaxonomic value as geochemical markers of fossil taxa. Nicholas et al. [7] have reported small amounts of well preserved fossil resin (amber) in association with the fossil flora within lignitic marls of the Grange Chine, Wessex Formation: Wealden Group (Barremian: Early Cretaceous) on the south west coast of the Isle of Wight. The lignitic marls are associated with fossiliferous green-gray mudstones that are characterized by abundant plant debris [8]. The locality is described in detail by Nicholas et al. [7]. Nicholas et al. [7], and later Seldon [9], have reported infrared spectra of amber from the Isle of Wight and on the basis of the results obtained have suggested familial affiliation with the extant families *Cupressaceae, Araucariaceae*, or *Taxodiaceae*. However, spectroscopic analyses of complex mixtures such as resins provide only a broad "fingerprint" and are, therefore, significantly less reliable than detailed molecular level characterization for chemotaxonomic purposes. The objective of this work was to attempt to determine possible paleobotanical sources of these ambers by using Pyrolysis-gas chromatography-mass spectrometry (Py-GC-MS) analyses.

## Experimental

Samples of amber from the Isle of Wight were obtained by request from the Dinosaur Isle Museum, Isle of Wight. Py-GC-MS analyses of the amber were performed as previously described [10]. Pyrolytic techniques for resin analysis require minimal sample (typically <500 μg/analysis), provide detailed molecular level data for individual products and, by variation of pyrolysis conditions, can provide information concerning both occluded low molecular weight components and macromolecular constituents of the sample [11]. For the studies described herein, samples were fractured, and small pieces (~200–400 μg) were pyrolyzed with tetramethylammonium hydroxide (TMAH) and the resulting products analyzed by gas chromatography mass spectrometry. Samples were pyrolyzed at temperatures of T_py _= 300 or 480°C to differentiate occluded volatile terpenes and macromolecular materials. Multiple analyses of individual blebs were performed and multiple discrete amber blebs were characterized to ensure that data generated are representative and to exclude the possibility of multiple discrete resins sources. Individual analytes are identified based on comparison of MS data with literature [12] and library data and in some cases, by comparison of MS results and chromatographic behavior with those of known standards.

## Results

**Figure 1:**
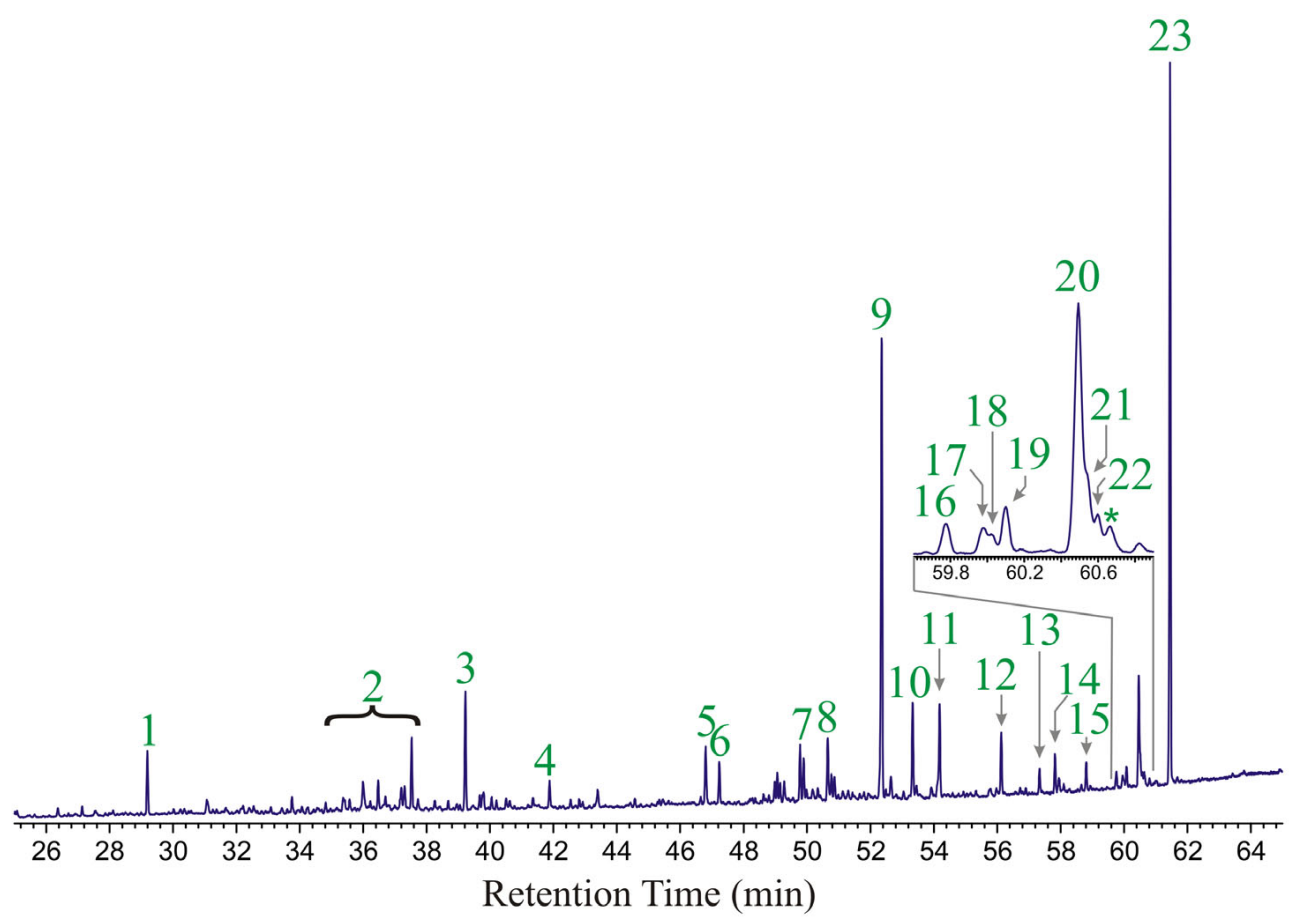
Distribution of products observed by Py-GC-MS (T_py _= 480°C) analysis of lower Cretaceous (Barremian) amber from the Isle of Wight. Structures of the observed analytes are illustrated below the chromatogram. Number of chromatographic peaks corresponds to numbering below individual structures. Enlargement of the region from 59.6–60.9 minutes is inset for clarity. * = Unassignable coeluting mixture.

**Figure 2:**
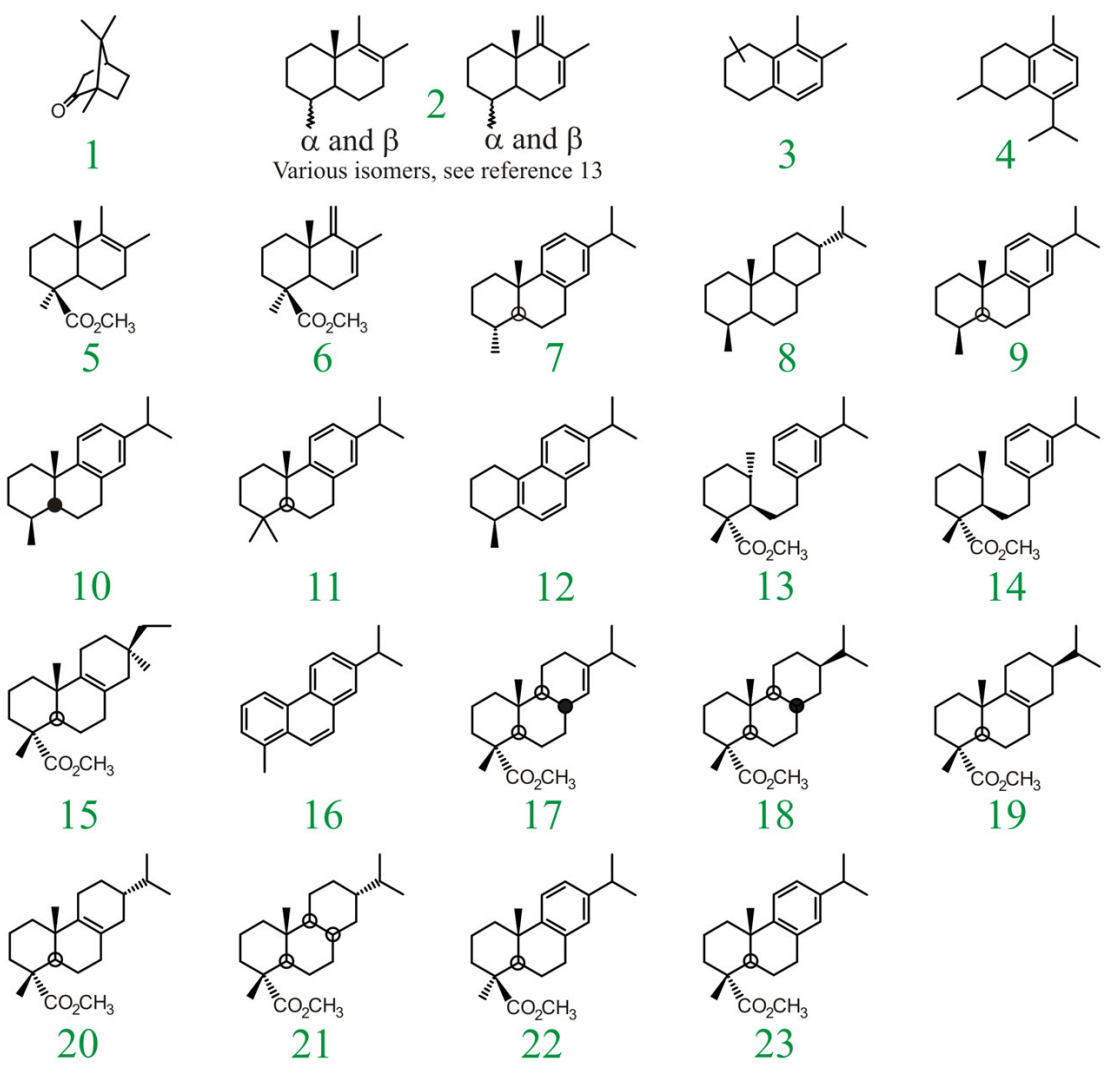
Structure Key. Numbering of individual structures corresponds with numbering of chromatographic peaks illustrated in Figure 1.

The results of Py-GC-MS analysis (T_py_= 480°C) of Isle of Wight amber are illustrated in Figure 1. Supporting data are also given in Additional File 1. Except as noted below in discussion of experiments carried out at different pyrolysis temperatures, all of the data generated in these analyses are identical to the results illustrated in Figure 1, indicating that all of the individual amber fragments analyzed are derived from a common botanical source. The structures of the products identified are illustrated in Figure 2.

The products observed in all analyses are dominated by abietane-type diterpenoids, especially dehydroabietic acid (23), 18-nor dehydroabietane (9) and lesser amounts of analogous and isomeric products. Small amounts of bicyclic products (5 and 6) and related decarboxylation products (2), characteristic of regular polylabdanoids [13-15], are also observed. Trace amounts of a single pimarane-type diterpenoid (15) are also observed. Diterpenoids of other structural families are apparently absent.

The distribution of intact diterpenoids observed in experiments carried out at T_py _= 300°C is indistinguishable from that observed in Figure 1. Under these conditions, macromolecular materials are not broken down into lower molecular weight volatile fragments and hence, are not represented in the observed analytes (i.e. compounds 2, 5, and 6 are absent in these data). The identity of the distributions of abietane-type terpenoids in the 480°C and 300°C data indicates that these compounds are present as occluded materials that are not bound into a macromolecular network.

## Discussion

Previous reports of analyses of Cretaceous ambers contain familiar, multi-class terpene distributions [10,16,17] and there is no reasonable geochemical route for conversion of other diterpenoids into the abietane-type structures observed in this distribution. There is also no geochemical evidence suggesting significant thermal alteration or excessive oxidation of these samples, and the depositional setting from which they were recovered suggests a very mild geologic history. Therefore, these data represent a unique chemotaxonomic signature of the original source resin.

Labdanoid- and abietane-type diterpenoids are common constituents of the resins of many extant conifer families but typically occur in conjunction with a variety of other types of diterpenoids. In modern resins, the exclusive presence of labdanoid- and abietane-type diterpenoids is an indicator of the *Pinaceae *[6,18-20]. *Pinaceae *resins are usually predominated by abietanoic acids and lack phenolic diterpenes such as ferruginol or sugiol [6]. These observations are consistent with the results presented here, wherein the Isle of Wight analyses are dominated by dehydroabietic acid and phenolic abietanes are absent. *Pinaceae *resins are also marked by an absence of tetracyclic terpenoids, e.g. kaurane or phyllocladane [6], also consistent with the data presented here.

By analogy with extant resins the observed distribution of diterpenes in these samples suggests a paleobotanical affinity with the *Pinaceae*. This assignment is supported by the minor presence of an early pinaceous species (*Pityites solmsii *(Seward) Seward) within the Wealdon deposits [4].

However, macrofossil evidence indicates that species belonging to the *Cheirolepidiace *were by far the dominant conifers contributing to these deposits. Phylogenetic relationships between the *Cheirolepidiaceae *and extant conifer families are uncertain [21]. To date, only one instance of possibly cheirolepidiaceous amber has been reported [22] and no rigorous geochemical analysis of amber unambiguously associated with the *Cheirolepidiace *has been described [16]. Data describing the extract of fossil leaves of *Frenelopsis alata *has been reported [23], showing that the extract contained many terpenoids associated with conifer resins, including some abietane type diterpenes. However, the relationship between the terpene composition of leaf extracts and resins is tenuous. Furthermore, the leaf extracts reported are from the cheirolepidiaceous genus *Frenelopsis*. The genera of *Cheirolepidiaceae *present in the Isle of Wight deposits are reported to be *Pseudofrenelopsis *and *Brachyphyllum*. Despite the similarity of the names assigned to these genera (*Frenelopsis *and *Pseudofrenelopsis*), these are distinct taxa and therefore, these data do not preclude the possibility that the resin chemistry of the *Cheirolepidiaceae *genera present in the Isle of Wight deposits may have been similar to the resin chemistry of the extant *Pinaceae*. Hence, the lack of geochemical data, as well as the dominance of the *Cheirolepidiaceae *within these deposits, precludes *a prioi *exclusion of this family as a potential source of these samples. Therefore, in the absence of additional data, the *Cheirolepidiaceae *must be included as a possible source for the samples characterized in the present study.

## Conclusion

Py-GC-MS analyses of early Cretaceous resin from the Isle of Wight, southern England, indicate that these resins are dominated nearly exclusively by abietane-type and labdanoid-type diterpenes. While this distribution provides a compelling argument for the assignment of these samples to the *Pinaceae*, the lack of geochemical data for the dominant conifers in the deposits, the *Cheirolepidiaceae*, precludes the exclusion of this family as a possible source of these samples. It is also not unreasonable to suggest that the *Pinaceae *may be under represented in the fossil record or that *Pityites solmsii *(Seward) Seward, the only well represented pinaceous species from the Isle of Wight, may have been a copious resin producer in the environment. Therefore, until clear data concerning the resin chemistry of the *Cheirolepidiaceae *is presented, the distribution presented here is equivocally assignable to either family. This work has demonstrated the use of resin chemistry in the Early Cretaceous, and these resins are the oldest resins to date to yield useful chemosystematic data.

## Supplementary Material

Additional file 1 GC-MS data for Isle of Wight amber. Interactive HTML version of data for Figures 1 and 2, including machine readable structure and MS data are given in Additional File 1.zip. These data are included to allow readers to access data underlying the structural assignments given. To access these data, download this file and unzip the compressed archive, ensuring that the embedded directory structure is preserved. Once uncompressed, simply open Index.html. Javascript must be enabled in your web browser in order to fully access these files. These files will also be available on line via the Geochemical Transactions web site in the near future. Click here for file
