# Supplementary figures and images for: The nature and fate of natural resins in the geosphere XIII: a probable pinaceous resin from the early Cretaceous (Barremian), Isle of Wight (part 2 of 2)
Source: Geochem Trans. 2008 Jan 29;9:3. doi: 10.1186/1467-4866-9-3 (PMC2265267; doi:10.1186/1467-4866-9-3)

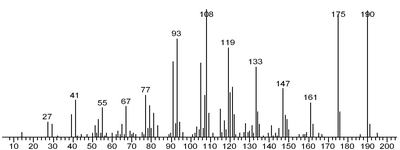

Supplement: Additional file 1 — GC-MS data for Isle of Wight amber. Interactive HTML version of data for Figures 1 and 2, including machine readable structure and MS data are given in Additional File 1.zip. These data are included to allow readers to access data underlying the structural assignments given. To access these data, download this file and unzip the compressed archive, ensuring that the embedded directory structure is preserved. Once uncompressed, simply open Index.html. Javascript must be enabled in your web browser in order to fully access these files. These files will also be available on line via the Geochemical Transactions web site in the near future. [file 1467-4866-9-3-S1.zip › Aditional File 1/Data1/MSData1/DS1MS3.png]

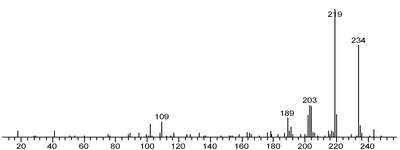

Supplement: Additional file 1 — GC-MS data for Isle of Wight amber. Interactive HTML version of data for Figures 1 and 2, including machine readable structure and MS data are given in Additional File 1.zip. These data are included to allow readers to access data underlying the structural assignments given. To access these data, download this file and unzip the compressed archive, ensuring that the embedded directory structure is preserved. Once uncompressed, simply open Index.html. Javascript must be enabled in your web browser in order to fully access these files. These files will also be available on line via the Geochemical Transactions web site in the near future. [file 1467-4866-9-3-S1.zip › Aditional File 1/Data1/MSData1/DS1MS30.png]

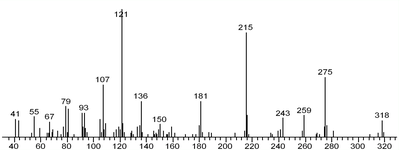

Supplement: Additional file 1 — GC-MS data for Isle of Wight amber. Interactive HTML version of data for Figures 1 and 2, including machine readable structure and MS data are given in Additional File 1.zip. These data are included to allow readers to access data underlying the structural assignments given. To access these data, download this file and unzip the compressed archive, ensuring that the embedded directory structure is preserved. Once uncompressed, simply open Index.html. Javascript must be enabled in your web browser in order to fully access these files. These files will also be available on line via the Geochemical Transactions web site in the near future. [file 1467-4866-9-3-S1.zip › Aditional File 1/Data1/MSData1/DS1MS31.png]

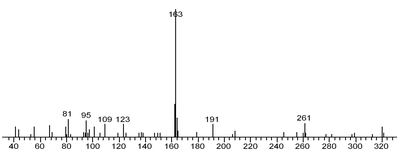

Supplement: Additional file 1 — GC-MS data for Isle of Wight amber. Interactive HTML version of data for Figures 1 and 2, including machine readable structure and MS data are given in Additional File 1.zip. These data are included to allow readers to access data underlying the structural assignments given. To access these data, download this file and unzip the compressed archive, ensuring that the embedded directory structure is preserved. Once uncompressed, simply open Index.html. Javascript must be enabled in your web browser in order to fully access these files. These files will also be available on line via the Geochemical Transactions web site in the near future. [file 1467-4866-9-3-S1.zip › Aditional File 1/Data1/MSData1/DS1MS32.png]

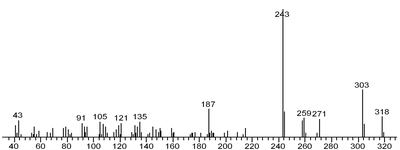

Supplement: Additional file 1 — GC-MS data for Isle of Wight amber. Interactive HTML version of data for Figures 1 and 2, including machine readable structure and MS data are given in Additional File 1.zip. These data are included to allow readers to access data underlying the structural assignments given. To access these data, download this file and unzip the compressed archive, ensuring that the embedded directory structure is preserved. Once uncompressed, simply open Index.html. Javascript must be enabled in your web browser in order to fully access these files. These files will also be available on line via the Geochemical Transactions web site in the near future. [file 1467-4866-9-3-S1.zip › Aditional File 1/Data1/MSData1/DS1MS33.png]

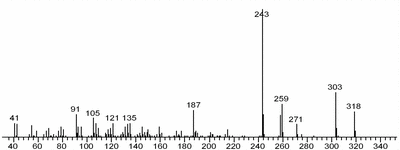

Supplement: Additional file 1 — GC-MS data for Isle of Wight amber. Interactive HTML version of data for Figures 1 and 2, including machine readable structure and MS data are given in Additional File 1.zip. These data are included to allow readers to access data underlying the structural assignments given. To access these data, download this file and unzip the compressed archive, ensuring that the embedded directory structure is preserved. Once uncompressed, simply open Index.html. Javascript must be enabled in your web browser in order to fully access these files. These files will also be available on line via the Geochemical Transactions web site in the near future. [file 1467-4866-9-3-S1.zip › Aditional File 1/Data1/MSData1/DS1MS34.png]

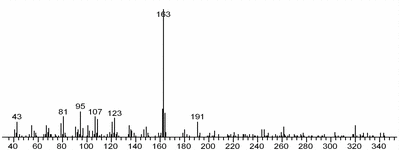

Supplement: Additional file 1 — GC-MS data for Isle of Wight amber. Interactive HTML version of data for Figures 1 and 2, including machine readable structure and MS data are given in Additional File 1.zip. These data are included to allow readers to access data underlying the structural assignments given. To access these data, download this file and unzip the compressed archive, ensuring that the embedded directory structure is preserved. Once uncompressed, simply open Index.html. Javascript must be enabled in your web browser in order to fully access these files. These files will also be available on line via the Geochemical Transactions web site in the near future. [file 1467-4866-9-3-S1.zip › Aditional File 1/Data1/MSData1/DS1MS35.png]

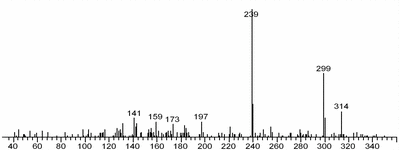

Supplement: Additional file 1 — GC-MS data for Isle of Wight amber. Interactive HTML version of data for Figures 1 and 2, including machine readable structure and MS data are given in Additional File 1.zip. These data are included to allow readers to access data underlying the structural assignments given. To access these data, download this file and unzip the compressed archive, ensuring that the embedded directory structure is preserved. Once uncompressed, simply open Index.html. Javascript must be enabled in your web browser in order to fully access these files. These files will also be available on line via the Geochemical Transactions web site in the near future. [file 1467-4866-9-3-S1.zip › Aditional File 1/Data1/MSData1/DS1MS36.png]

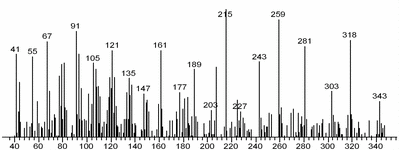

Supplement: Additional file 1 — GC-MS data for Isle of Wight amber. Interactive HTML version of data for Figures 1 and 2, including machine readable structure and MS data are given in Additional File 1.zip. These data are included to allow readers to access data underlying the structural assignments given. To access these data, download this file and unzip the compressed archive, ensuring that the embedded directory structure is preserved. Once uncompressed, simply open Index.html. Javascript must be enabled in your web browser in order to fully access these files. These files will also be available on line via the Geochemical Transactions web site in the near future. [file 1467-4866-9-3-S1.zip › Aditional File 1/Data1/MSData1/DS1MS37.png]

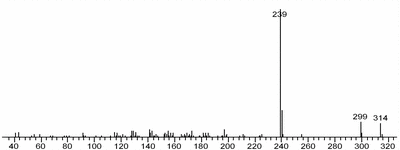

Supplement: Additional file 1 — GC-MS data for Isle of Wight amber. Interactive HTML version of data for Figures 1 and 2, including machine readable structure and MS data are given in Additional File 1.zip. These data are included to allow readers to access data underlying the structural assignments given. To access these data, download this file and unzip the compressed archive, ensuring that the embedded directory structure is preserved. Once uncompressed, simply open Index.html. Javascript must be enabled in your web browser in order to fully access these files. These files will also be available on line via the Geochemical Transactions web site in the near future. [file 1467-4866-9-3-S1.zip › Aditional File 1/Data1/MSData1/DS1MS38.png]

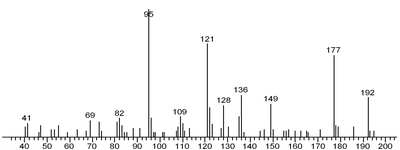

Supplement: Additional file 1 — GC-MS data for Isle of Wight amber. Interactive HTML version of data for Figures 1 and 2, including machine readable structure and MS data are given in Additional File 1.zip. These data are included to allow readers to access data underlying the structural assignments given. To access these data, download this file and unzip the compressed archive, ensuring that the embedded directory structure is preserved. Once uncompressed, simply open Index.html. Javascript must be enabled in your web browser in order to fully access these files. These files will also be available on line via the Geochemical Transactions web site in the near future. [file 1467-4866-9-3-S1.zip › Aditional File 1/Data1/MSData1/DS1MS4.png]

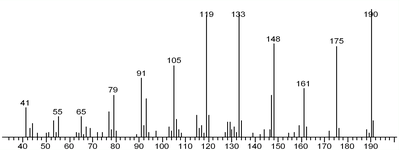

Supplement: Additional file 1 — GC-MS data for Isle of Wight amber. Interactive HTML version of data for Figures 1 and 2, including machine readable structure and MS data are given in Additional File 1.zip. These data are included to allow readers to access data underlying the structural assignments given. To access these data, download this file and unzip the compressed archive, ensuring that the embedded directory structure is preserved. Once uncompressed, simply open Index.html. Javascript must be enabled in your web browser in order to fully access these files. These files will also be available on line via the Geochemical Transactions web site in the near future. [file 1467-4866-9-3-S1.zip › Aditional File 1/Data1/MSData1/DS1MS5.png]

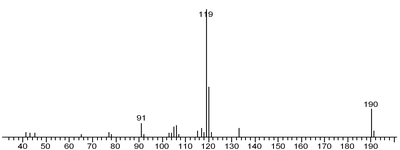

Supplement: Additional file 1 — GC-MS data for Isle of Wight amber. Interactive HTML version of data for Figures 1 and 2, including machine readable structure and MS data are given in Additional File 1.zip. These data are included to allow readers to access data underlying the structural assignments given. To access these data, download this file and unzip the compressed archive, ensuring that the embedded directory structure is preserved. Once uncompressed, simply open Index.html. Javascript must be enabled in your web browser in order to fully access these files. These files will also be available on line via the Geochemical Transactions web site in the near future. [file 1467-4866-9-3-S1.zip › Aditional File 1/Data1/MSData1/DS1MS6.png]

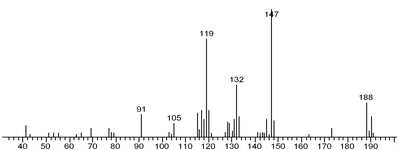

Supplement: Additional file 1 — GC-MS data for Isle of Wight amber. Interactive HTML version of data for Figures 1 and 2, including machine readable structure and MS data are given in Additional File 1.zip. These data are included to allow readers to access data underlying the structural assignments given. To access these data, download this file and unzip the compressed archive, ensuring that the embedded directory structure is preserved. Once uncompressed, simply open Index.html. Javascript must be enabled in your web browser in order to fully access these files. These files will also be available on line via the Geochemical Transactions web site in the near future. [file 1467-4866-9-3-S1.zip › Aditional File 1/Data1/MSData1/DS1MS7.png]

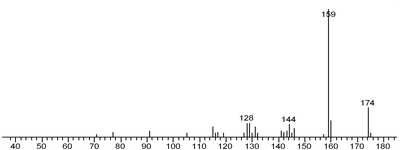

Supplement: Additional file 1 — GC-MS data for Isle of Wight amber. Interactive HTML version of data for Figures 1 and 2, including machine readable structure and MS data are given in Additional File 1.zip. These data are included to allow readers to access data underlying the structural assignments given. To access these data, download this file and unzip the compressed archive, ensuring that the embedded directory structure is preserved. Once uncompressed, simply open Index.html. Javascript must be enabled in your web browser in order to fully access these files. These files will also be available on line via the Geochemical Transactions web site in the near future. [file 1467-4866-9-3-S1.zip › Aditional File 1/Data1/MSData1/DS1MS8.png]

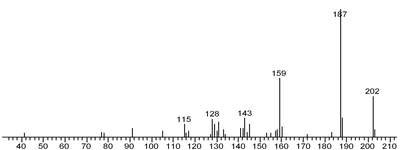

Supplement: Additional file 1 — GC-MS data for Isle of Wight amber. Interactive HTML version of data for Figures 1 and 2, including machine readable structure and MS data are given in Additional File 1.zip. These data are included to allow readers to access data underlying the structural assignments given. To access these data, download this file and unzip the compressed archive, ensuring that the embedded directory structure is preserved. Once uncompressed, simply open Index.html. Javascript must be enabled in your web browser in order to fully access these files. These files will also be available on line via the Geochemical Transactions web site in the near future. [file 1467-4866-9-3-S1.zip › Aditional File 1/Data1/MSData1/DS1MS9.png]

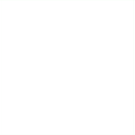

Supplement: Additional file 1 — GC-MS data for Isle of Wight amber. Interactive HTML version of data for Figures 1 and 2, including machine readable structure and MS data are given in Additional File 1.zip. These data are included to allow readers to access data underlying the structural assignments given. To access these data, download this file and unzip the compressed archive, ensuring that the embedded directory structure is preserved. Once uncompressed, simply open Index.html. Javascript must be enabled in your web browser in order to fully access these files. These files will also be available on line via the Geochemical Transactions web site in the near future. [file 1467-4866-9-3-S1.zip › Aditional File 1/Structures/Structure0.png]

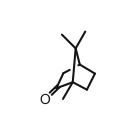

Supplement: Additional file 1 — GC-MS data for Isle of Wight amber. Interactive HTML version of data for Figures 1 and 2, including machine readable structure and MS data are given in Additional File 1.zip. These data are included to allow readers to access data underlying the structural assignments given. To access these data, download this file and unzip the compressed archive, ensuring that the embedded directory structure is preserved. Once uncompressed, simply open Index.html. Javascript must be enabled in your web browser in order to fully access these files. These files will also be available on line via the Geochemical Transactions web site in the near future. [file 1467-4866-9-3-S1.zip › Aditional File 1/Structures/Structure1.png]

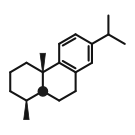

Supplement: Additional file 1 — GC-MS data for Isle of Wight amber. Interactive HTML version of data for Figures 1 and 2, including machine readable structure and MS data are given in Additional File 1.zip. These data are included to allow readers to access data underlying the structural assignments given. To access these data, download this file and unzip the compressed archive, ensuring that the embedded directory structure is preserved. Once uncompressed, simply open Index.html. Javascript must be enabled in your web browser in order to fully access these files. These files will also be available on line via the Geochemical Transactions web site in the near future. [file 1467-4866-9-3-S1.zip › Aditional File 1/Structures/Structure10.png]

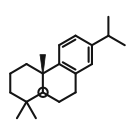

Supplement: Additional file 1 — GC-MS data for Isle of Wight amber. Interactive HTML version of data for Figures 1 and 2, including machine readable structure and MS data are given in Additional File 1.zip. These data are included to allow readers to access data underlying the structural assignments given. To access these data, download this file and unzip the compressed archive, ensuring that the embedded directory structure is preserved. Once uncompressed, simply open Index.html. Javascript must be enabled in your web browser in order to fully access these files. These files will also be available on line via the Geochemical Transactions web site in the near future. [file 1467-4866-9-3-S1.zip › Aditional File 1/Structures/Structure11.png]

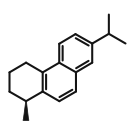

Supplement: Additional file 1 — GC-MS data for Isle of Wight amber. Interactive HTML version of data for Figures 1 and 2, including machine readable structure and MS data are given in Additional File 1.zip. These data are included to allow readers to access data underlying the structural assignments given. To access these data, download this file and unzip the compressed archive, ensuring that the embedded directory structure is preserved. Once uncompressed, simply open Index.html. Javascript must be enabled in your web browser in order to fully access these files. These files will also be available on line via the Geochemical Transactions web site in the near future. [file 1467-4866-9-3-S1.zip › Aditional File 1/Structures/Structure12.png]

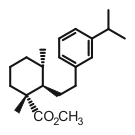

Supplement: Additional file 1 — GC-MS data for Isle of Wight amber. Interactive HTML version of data for Figures 1 and 2, including machine readable structure and MS data are given in Additional File 1.zip. These data are included to allow readers to access data underlying the structural assignments given. To access these data, download this file and unzip the compressed archive, ensuring that the embedded directory structure is preserved. Once uncompressed, simply open Index.html. Javascript must be enabled in your web browser in order to fully access these files. These files will also be available on line via the Geochemical Transactions web site in the near future. [file 1467-4866-9-3-S1.zip › Aditional File 1/Structures/Structure13.png]

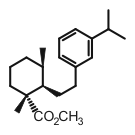

Supplement: Additional file 1 — GC-MS data for Isle of Wight amber. Interactive HTML version of data for Figures 1 and 2, including machine readable structure and MS data are given in Additional File 1.zip. These data are included to allow readers to access data underlying the structural assignments given. To access these data, download this file and unzip the compressed archive, ensuring that the embedded directory structure is preserved. Once uncompressed, simply open Index.html. Javascript must be enabled in your web browser in order to fully access these files. These files will also be available on line via the Geochemical Transactions web site in the near future. [file 1467-4866-9-3-S1.zip › Aditional File 1/Structures/Structure14.png]

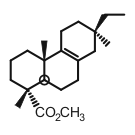

Supplement: Additional file 1 — GC-MS data for Isle of Wight amber. Interactive HTML version of data for Figures 1 and 2, including machine readable structure and MS data are given in Additional File 1.zip. These data are included to allow readers to access data underlying the structural assignments given. To access these data, download this file and unzip the compressed archive, ensuring that the embedded directory structure is preserved. Once uncompressed, simply open Index.html. Javascript must be enabled in your web browser in order to fully access these files. These files will also be available on line via the Geochemical Transactions web site in the near future. [file 1467-4866-9-3-S1.zip › Aditional File 1/Structures/Structure15.png]

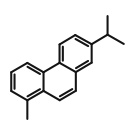

Supplement: Additional file 1 — GC-MS data for Isle of Wight amber. Interactive HTML version of data for Figures 1 and 2, including machine readable structure and MS data are given in Additional File 1.zip. These data are included to allow readers to access data underlying the structural assignments given. To access these data, download this file and unzip the compressed archive, ensuring that the embedded directory structure is preserved. Once uncompressed, simply open Index.html. Javascript must be enabled in your web browser in order to fully access these files. These files will also be available on line via the Geochemical Transactions web site in the near future. [file 1467-4866-9-3-S1.zip › Aditional File 1/Structures/Structure16.png]

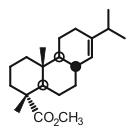

Supplement: Additional file 1 — GC-MS data for Isle of Wight amber. Interactive HTML version of data for Figures 1 and 2, including machine readable structure and MS data are given in Additional File 1.zip. These data are included to allow readers to access data underlying the structural assignments given. To access these data, download this file and unzip the compressed archive, ensuring that the embedded directory structure is preserved. Once uncompressed, simply open Index.html. Javascript must be enabled in your web browser in order to fully access these files. These files will also be available on line via the Geochemical Transactions web site in the near future. [file 1467-4866-9-3-S1.zip › Aditional File 1/Structures/Structure17.png]

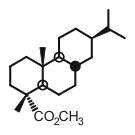

Supplement: Additional file 1 — GC-MS data for Isle of Wight amber. Interactive HTML version of data for Figures 1 and 2, including machine readable structure and MS data are given in Additional File 1.zip. These data are included to allow readers to access data underlying the structural assignments given. To access these data, download this file and unzip the compressed archive, ensuring that the embedded directory structure is preserved. Once uncompressed, simply open Index.html. Javascript must be enabled in your web browser in order to fully access these files. These files will also be available on line via the Geochemical Transactions web site in the near future. [file 1467-4866-9-3-S1.zip › Aditional File 1/Structures/Structure18.png]

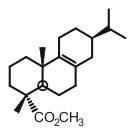

Supplement: Additional file 1 — GC-MS data for Isle of Wight amber. Interactive HTML version of data for Figures 1 and 2, including machine readable structure and MS data are given in Additional File 1.zip. These data are included to allow readers to access data underlying the structural assignments given. To access these data, download this file and unzip the compressed archive, ensuring that the embedded directory structure is preserved. Once uncompressed, simply open Index.html. Javascript must be enabled in your web browser in order to fully access these files. These files will also be available on line via the Geochemical Transactions web site in the near future. [file 1467-4866-9-3-S1.zip › Aditional File 1/Structures/Structure19.png]

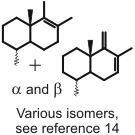

Supplement: Additional file 1 — GC-MS data for Isle of Wight amber. Interactive HTML version of data for Figures 1 and 2, including machine readable structure and MS data are given in Additional File 1.zip. These data are included to allow readers to access data underlying the structural assignments given. To access these data, download this file and unzip the compressed archive, ensuring that the embedded directory structure is preserved. Once uncompressed, simply open Index.html. Javascript must be enabled in your web browser in order to fully access these files. These files will also be available on line via the Geochemical Transactions web site in the near future. [file 1467-4866-9-3-S1.zip › Aditional File 1/Structures/Structure2.png]

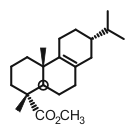

Supplement: Additional file 1 — GC-MS data for Isle of Wight amber. Interactive HTML version of data for Figures 1 and 2, including machine readable structure and MS data are given in Additional File 1.zip. These data are included to allow readers to access data underlying the structural assignments given. To access these data, download this file and unzip the compressed archive, ensuring that the embedded directory structure is preserved. Once uncompressed, simply open Index.html. Javascript must be enabled in your web browser in order to fully access these files. These files will also be available on line via the Geochemical Transactions web site in the near future. [file 1467-4866-9-3-S1.zip › Aditional File 1/Structures/Structure20.png]

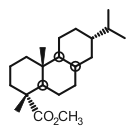

Supplement: Additional file 1 — GC-MS data for Isle of Wight amber. Interactive HTML version of data for Figures 1 and 2, including machine readable structure and MS data are given in Additional File 1.zip. These data are included to allow readers to access data underlying the structural assignments given. To access these data, download this file and unzip the compressed archive, ensuring that the embedded directory structure is preserved. Once uncompressed, simply open Index.html. Javascript must be enabled in your web browser in order to fully access these files. These files will also be available on line via the Geochemical Transactions web site in the near future. [file 1467-4866-9-3-S1.zip › Aditional File 1/Structures/Structure21.png]

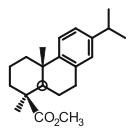

Supplement: Additional file 1 — GC-MS data for Isle of Wight amber. Interactive HTML version of data for Figures 1 and 2, including machine readable structure and MS data are given in Additional File 1.zip. These data are included to allow readers to access data underlying the structural assignments given. To access these data, download this file and unzip the compressed archive, ensuring that the embedded directory structure is preserved. Once uncompressed, simply open Index.html. Javascript must be enabled in your web browser in order to fully access these files. These files will also be available on line via the Geochemical Transactions web site in the near future. [file 1467-4866-9-3-S1.zip › Aditional File 1/Structures/Structure22.png]

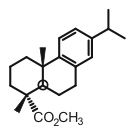

Supplement: Additional file 1 — GC-MS data for Isle of Wight amber. Interactive HTML version of data for Figures 1 and 2, including machine readable structure and MS data are given in Additional File 1.zip. These data are included to allow readers to access data underlying the structural assignments given. To access these data, download this file and unzip the compressed archive, ensuring that the embedded directory structure is preserved. Once uncompressed, simply open Index.html. Javascript must be enabled in your web browser in order to fully access these files. These files will also be available on line via the Geochemical Transactions web site in the near future. [file 1467-4866-9-3-S1.zip › Aditional File 1/Structures/Structure23.png]

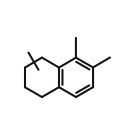

Supplement: Additional file 1 — GC-MS data for Isle of Wight amber. Interactive HTML version of data for Figures 1 and 2, including machine readable structure and MS data are given in Additional File 1.zip. These data are included to allow readers to access data underlying the structural assignments given. To access these data, download this file and unzip the compressed archive, ensuring that the embedded directory structure is preserved. Once uncompressed, simply open Index.html. Javascript must be enabled in your web browser in order to fully access these files. These files will also be available on line via the Geochemical Transactions web site in the near future. [file 1467-4866-9-3-S1.zip › Aditional File 1/Structures/Structure3.png]

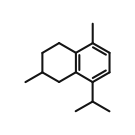

Supplement: Additional file 1 — GC-MS data for Isle of Wight amber. Interactive HTML version of data for Figures 1 and 2, including machine readable structure and MS data are given in Additional File 1.zip. These data are included to allow readers to access data underlying the structural assignments given. To access these data, download this file and unzip the compressed archive, ensuring that the embedded directory structure is preserved. Once uncompressed, simply open Index.html. Javascript must be enabled in your web browser in order to fully access these files. These files will also be available on line via the Geochemical Transactions web site in the near future. [file 1467-4866-9-3-S1.zip › Aditional File 1/Structures/Structure4.png]

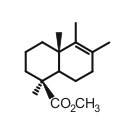

Supplement: Additional file 1 — GC-MS data for Isle of Wight amber. Interactive HTML version of data for Figures 1 and 2, including machine readable structure and MS data are given in Additional File 1.zip. These data are included to allow readers to access data underlying the structural assignments given. To access these data, download this file and unzip the compressed archive, ensuring that the embedded directory structure is preserved. Once uncompressed, simply open Index.html. Javascript must be enabled in your web browser in order to fully access these files. These files will also be available on line via the Geochemical Transactions web site in the near future. [file 1467-4866-9-3-S1.zip › Aditional File 1/Structures/Structure5.png]

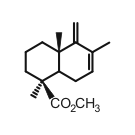

Supplement: Additional file 1 — GC-MS data for Isle of Wight amber. Interactive HTML version of data for Figures 1 and 2, including machine readable structure and MS data are given in Additional File 1.zip. These data are included to allow readers to access data underlying the structural assignments given. To access these data, download this file and unzip the compressed archive, ensuring that the embedded directory structure is preserved. Once uncompressed, simply open Index.html. Javascript must be enabled in your web browser in order to fully access these files. These files will also be available on line via the Geochemical Transactions web site in the near future. [file 1467-4866-9-3-S1.zip › Aditional File 1/Structures/Structure6.png]

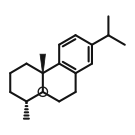

Supplement: Additional file 1 — GC-MS data for Isle of Wight amber. Interactive HTML version of data for Figures 1 and 2, including machine readable structure and MS data are given in Additional File 1.zip. These data are included to allow readers to access data underlying the structural assignments given. To access these data, download this file and unzip the compressed archive, ensuring that the embedded directory structure is preserved. Once uncompressed, simply open Index.html. Javascript must be enabled in your web browser in order to fully access these files. These files will also be available on line via the Geochemical Transactions web site in the near future. [file 1467-4866-9-3-S1.zip › Aditional File 1/Structures/Structure7.png]

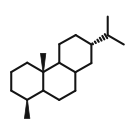

Supplement: Additional file 1 — GC-MS data for Isle of Wight amber. Interactive HTML version of data for Figures 1 and 2, including machine readable structure and MS data are given in Additional File 1.zip. These data are included to allow readers to access data underlying the structural assignments given. To access these data, download this file and unzip the compressed archive, ensuring that the embedded directory structure is preserved. Once uncompressed, simply open Index.html. Javascript must be enabled in your web browser in order to fully access these files. These files will also be available on line via the Geochemical Transactions web site in the near future. [file 1467-4866-9-3-S1.zip › Aditional File 1/Structures/Structure8.png]

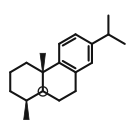

Supplement: Additional file 1 — GC-MS data for Isle of Wight amber. Interactive HTML version of data for Figures 1 and 2, including machine readable structure and MS data are given in Additional File 1.zip. These data are included to allow readers to access data underlying the structural assignments given. To access these data, download this file and unzip the compressed archive, ensuring that the embedded directory structure is preserved. Once uncompressed, simply open Index.html. Javascript must be enabled in your web browser in order to fully access these files. These files will also be available on line via the Geochemical Transactions web site in the near future. [file 1467-4866-9-3-S1.zip › Aditional File 1/Structures/Structure9.png]
